# Supplementary material for: Thriving in Wetlands: Ecophysiology of the Spiral-Shaped Methanotroph Methylospira mobilis as Revealed by the Complete Genome Sequence
Source: Microorganisms. 2019 Dec 11;7(12):683. doi: 10.3390/microorganisms7120683 (PMC6956133; doi:10.3390/microorganisms7120683)
Supplement: Supplementary file 1 [file microorganisms-07-00683-s001.pdf]

# Supplementary Material

# Thriving in wetlands: Ecophysiology of the spiral-shaped methanotroph *Methylospira mobilis* as revealed by the complete genome sequence

Igor Y. Oshkin[1], Kirill K. Miroshnikov[1], Olga V. Danilova[1], Anna Hakobyan[2], Werner Liesack[2], Svetlana N. Dedysh[1]

[1]Winogradsky Institute of Microbiology, Research Center of Biotechnology of the Russian Academy of Sciences, Moscow 119071, Russia;

[2]Max-Planck-Institut für terrestrische Mikrobiologie, D-35043 Marburg, Germany.

Correspondence: Svetlana N. Dedysh

Tel: 7 (499) 135 0591. Fax: 7 (499) 135 6530. Email: dedysh@mail.ru

**Running title**: Genome analysis of a spiral-shaped methanotroph.

**Supplementary Table S1**. Methylotrophy genes identified in the genomes *Methylococcus capsulatus* Bath and *Methylospira mobilis* Shm1 Positive detection of a homologue was based on annotations in Genbank, and on BLAST searches against NCBI and Uniprot databases.

| Gene name | Gene symbol | *M. capsulatus* | Coordinate 1 | Coordinate 2 | *M. mobilis* | Coordinate 1 | Coordinate 2 |
|---|---|---|---|---|---|---|---|
| **Methane oxidation** | | | | | | | |
| particulate methane monooxygenase (pMMO), subunit C | pmoC | MCA1798 | 1928445 | 1929227 | F6R98_01470 | 388598 | 389377 |
| | | MCA2855 | 3048914 | 3049696 | F6R98_16065 | 3544488 | 3543709 |
| particulate methane monooxygenase (pMMO), subunit A | pmoA | MCA1797 | 1927548 | 1928291 | F6R98_01475 | 389539 | 390282 |
| | | MCA2854 | 3048017 | 3048760 | F6R98_16060 | 3543547 | 3542804 |
| particulate methane monooxygenase (pMMO), subunit B | pmoB | MCA1796 | 1926197 | 1927441 | F6R98_01480 | 390434 | 391681 |
| | | MCA2853 | 3046666 | 3047910 | F6R98_16055 | 3542652 | 3541405 |
| soluble methane monooxygenase (sMMO), subunit alpha | *mmoX* | MCA1194 | 1252141 | 1253724 | F6R98_10915 | 2421999 | 2421999 |
| soluble methane monooxygenase (sMMO), subunit beta | *mmoY* | MCA1195 | 1253805 | 1254974 | F6R98_10910 | 2421925 | 2420756 |
| soluble methane monooxygenase (sMMO), regulatory component | *mmoB* | MCA1196 | 1254991 | 1255416 | F6R98_10905 | 2420745 | 2420317 |
| soluble methane monooxygenase (sMMO), subunit gamma | *mmoZ* | MCA1198 | 1256767 | 1257279 | F6R98_10900 | 2420303 | 2419797 |
| **Methanol oxidation** | | | | | | | |
| PQQ-dependent methanol dehydrogenase (MDH), large subunit | *mxaF* | MCA0779 | 823278 | 825083 | F6R98_08155 | 1857763 | 1859568 |
| periplasmic protein, probable ABC transporter substrate-binding protein | *mxaJ* | MCA0780 | 825441 | 826013 | F6R98_08160 | 1859625 | 1860473 |
| periplasmic c-type cytochrome, primary electron acceptor from PQQdependent | mxaG | MCA0781 | 826019 | 826498 | F6R98_08165 | 1860484 | 1860963 |
| PQQ-dependent methanol dehydrogenase (MDH), small subunit | *mxaI* | MCA0782 | 826540 | 826824 | F6R98_08170 | 1860991 | 1861281 |
| ATPase, P-loop NTPase family | *mxaR* | MCA0783 | 826988 | 828007 | F6R98_08175 | 1861343 | 1862368 |
| a von Willebrand factor type A (vWA) domain involved in methanol oxidation | mxaS | MCA0784 | 828004 | 828846 | F6R98_08180 | 1862365 | 1863210 |

| | | | | | | | |
|---|---|---|---|---|---|---|---|
| essential for Ca2+ insertion into MDH | *mxaA* | MCA0785 | 829035 | 829907 | F6R98_08185 | 1863207 | 1864121 |
| essential for Ca2+ insertion into MDH | *mxaC* | MCA0786 | 829904 | 830887 | F6R98_08190 | 1864118 | 1865116 |
| essential for Ca2+ insertion into MDH | *mxaK* | MCA0787 | 830929 | 831447 | F6R98_08195 | 1865101 | 1865667 |
| essential for Ca2+ insertion into MDH | *mxaL* | MCA0788 | 831444 | 832415 | F6R98_08200 | 1865664 | 1866686 |
| essential for Ca2+ insertion into MDH | *mxaD* | MCA0789 | 832434 | 832958 | F6R98_08205 | 1866683 | 1867207 |
| unknown | *mxaE* | MCA1279 | 1363415 | 1364404 | F6R98_08210 | 1867211 | 1868185 |
| transcriptional regulator | *mxaB* | MCA0777 | 821027 | 821674 | F6R98_08220 | 1868579 | 1869226 |
| coenzyme PQQ biosynthesis | pqqA | MCA1445.1 | 1538132 | 1538206 | F6R98_04695 | 1089243 | 1089317 |
| | | | | | F6R98_08215 | 1868278 | 1868352 |
| coenzyme PQQ biosynthesis | *pqqB* | MCA1446 | 1538293 | 1539207 | F6R98_08475 | 1932924 | 1933838 |
| coenzyme PQQ biosynthesis | *pqqC* | MCA1447 | 1539216 | 1539941 | F6R98_08480 | 1933898 | 1934623 |
| coenzyme PQQ biosynthesis | *pqqD* | MCA1448 | 1539947 | 1540228 | F6R98_08485 | 1934637 | 1934909 |
| coenzyme PQQ biosynthesis | *pqqE* | MCA1449 | 1540197 | 1541318 | F6R98_08490 | 1934887 | 1936011 |
| coenzyme PQQ biosynthesis | *pqqF* | MCA0063 | 64477 | 65856 | F6R98_11435 | 2541975 | 2540590 |
| coenzyme PQQ biosynthesis | *pqqG* | MCA0064 | 65846 | 67171 | F6R98_11430 | 2540600 | 2539272 |
| xoxF | xoxF | MCA0299 | 293402 | 295261 | F6R98_11505 | 2556553 | 2558418 |
| | | | | | F6R98_16780 | 3702340 | 3700475 |
| **C1 transfer / formaldehyde oxidation** | | | | | | | |
| formaldehyde activating enzyme | fae | MCA2778 | 2970552 | 2970911 | F6R98_15000 | 3305441 | 3304929 |
| | | MCA2866 | 3060088 | 3060600 | F6R98_19005 | 4173806 | 4173294 |
| | | | | | F6R98_04980 | 1157466 | 1156951 |
| methylene-tetrahydromethanopterin (H4MPT) dehydrogenase | mtdB | MCA3018 | 3201649 | 3202554 | F6R98_14510 | 3200473 | 3201384 |
| | | MCA3019 | 3202646 | 3203527 | F6R98_14515 | 3201733 | 3202647 |
| formyltransferase/hydrolase complex, alpha subunit | *fhcA* | MCA2859 | 3051796 | 3053463 | F6R98_03260 | 784596 | 786260 |
| formyltransferase/hydrolase complex, beta subunit | *fhcB* | MCA2860 | 3053492 | 3054763 | F6R98_03255 | 783305 | 784588 |
| formyltransferase/hydrolase complex, gamma subunit | *fhcC* | MCA2857 | 3050056 | 3050868 | F6R98_03270 | 787178 | 787996 |
| formyltransferase/hydrolase complex, delta subunit | *fhcD* | MCA2858 | 3050903 | 3051799 | F6R98_03265 | 786257 | 787153 |

| | | | | | | | |
|---|---|---|---|---|---|---|---|
| methylene-H4MPT /methylene-tetrahydrofolate (H4F) dehydrogenase | *mtdA* | MCA0508 | 521192 | 522052 | F6R98_09875 | 2223245 | 2224111 |
| methenyl-H4F cyclohydrolase | *fch* | MCA2863 | 3057182 | 3058177 | F6R98_19020 | 4176773 | 4175778 |
| dihydromethanopterin reductase | *dmrA* | Absent | | | Absent | | |
| formyl-H4F ligase | *ftfL* | MCA2219 | 2396781 | 2398454 | F6R98_03760 | 889085 | 890755 |
| 2-amino-4-hydroxy-6-hydroxymethyl-7,8-dihydropterin pyrophosphokinase | folk | MCA2313 | 2507044 | 2507541 | F6R98_18580 | 4092196 | 4092702 |
| | | MCA2987 | 3161584 | 3162078 | | | |
| dihydroneopterin aldolase | *folB* | MCA2988 | 3162071 | 3162436 | F6R98_18575 | 4091843 | 4092199 |
| dihydroneopterin synthase | *folP* | MCA2317 | 2509686 | 2510570 | F6R98_05160 | 1189782 | 1190627 |
| GTP cyclohydrolase | *folE* | MCA1670 | 1773965 | 1774522 | F6R98_12615 | 2795380 | 2795925 |
| dihydrofolate synthase | *folC* | MCA2492 | 2673335 | 2674630 | F6R98_08020 | 1828425 | 1829702 |
| methenyl-H4MPT cyclohydrolase | *mch* | MCA2863 | 3057182 | 3058177 | F6R98_19020 | 4175778 | 4176773 |
| dihydrofolate reductase | *dfrA* | MCA2729 | 2922594 | 2923112 | F6R98_05325 | 1223823 | 1224311 |
| **Formate oxidation** | | | | | | | |
| formate dehydrogenase, alpha subunit | fdhA | MCA1210 | 1271086 | 1274286 | F6R98_12575 | 2784595 | 2787438 |
| | | MCA1391 | 1474796 | 1477639 | | | |
| | | MCA2576 | 2758784 | 2761561 | | | |
| formate dehydrogenase, beta subunit | *fdhB* | MCA1392 | 1477696 | 1479246 | F6R98_12580 | 2787497 | 2789050 |
| formate dehydrogenase, gamma subunit | fdhC | MCA120 | 1269579 | 1270214 | F6R98_12585 | 2789047 | 2789523 |
| | | MCA1393 | 1479243 | 1479722 | | | |
| formate dehydrogenase, delta subunit | *fdhD* | MCA1389 | 1473618 | 1473851 | F6R98_12565 | 2782970 | 2783179 |
| NAD-dependent formate dehydrogenase | *fdh* | Absent | | | F6R98_01730 | 440807 | 442012 |
| **C1-assimilation: ribulose monophosphate pathway** | | | | | | | |
| 3-hexulose-6-phosphate synthase | hxlA | MCA3043 | 3230263 | 3230910 | F6R98_00315 | 76334 | 76993 |
| | | MCA3049 | 3236945 | 3237592 | F6R98_00325 | 77632 | 78279 |
| 6-phospho-3-hexuloisomerase | hxlB | MCA3044 | 3230923 | 3231456 | F6R98_00320 | 76995 | 77528 |
| | | MCA3050 | 3237605 | 3238138 | | | |
| Pyrophosphate-dependent fructose 6-phosphate-1-kinase (EC 2.7.1.90) | pfp | MCA1251 | 1330927 | 1332189 | F6R98_02840 | 679990 | 681249 |
| Fructose-bisphosphate aldolase class II | fbaA | MCA3041 | 3228215 | 3229279 | F6R98_00290 | 71340 | 72404 |

| | | | | | | | |
|---|---|---|---|---|---|---|---|
| **C1-assimilation: serine cycle** | | | | | | | |
| serine-glyoxylate aminotransferase | *sga* | MCA1406 | 1492959 | 1494146 | F6R98_06335 | 1450866 | 1452056 |
| hydroxypyruvate reductase | *hpr* | MCA1407 | 1494241 | 1495212 | F6R98_01730 | 440807 | 442012 |
| glycerate kinase | *gck* | MCA1408 | 1495321 | 1496187 | Absent | | |
| enolase | eno | MCA1933 | 2080068 | 2081333 | F6R98_16530 | 3645336 | 3644053 |
| | | MCA2515 | 2693051 | 2694334 | | | |
| phosphoenolpyruvate carboxylase | *ppcA* | Absent | | | F6R98_13095 | 2894412 | 2891602 |
| malate dehydrogenase | *mdh* | MCA0610 | 645424 | 646401 | F6R98_13210 | 2917209 | 2916232 |
| malate thiokinase, alpha subunit | *mtkA* | MCA1741 | 1858803 | 1859705 | F6R98_20180 | 4432178 | 4433065 |
| malate thiokinase, beta subunit | *mtkB* | MCA1740 | 1857628 | 1858797 | F6R98_20175 | 4431003 | 4432175 |
| malyl-CoA lyase | *mcl* | MCA1739 | 1856611 | 1857573 | F6R98_20170 | 4429946 | 4430911 |
| serine hydroxymethyltrasferase | *glyA* | MCA1660 | 1762420 | 1763676 | F6R98_12550 | 2781455 | 2780199 |
| **Calvin cycle** | | | | | | | |
| Phosphoribulokinase | prk | MCA3051 | 3238358 | 3239236 | F6R98_00300 | 73273 | 74169 |
| Ribulose bisphosphate carboxylase | cbbl | MCA2743 | 2934677 | 2936098 | F6R98_15900 | 3503068 | 3504447 |
| Phosphoglycerate kinase | pgk | MCA2021 | 2169482 | 2170657 | F6R98_11375 | 2528550 | 2527369 |
| glyceraldehyde 3-phosphate dehydrogenase | gapA | MCA2598 | 2783826 | 2784836 | F6R98_18320 | 4036685 | 4035675 |
| Fructose-bisphosphate aldolase class II | fbaA | MCA3041 | 3228215 | 3229279 | F6R98_00290 | 71340 | 72404 |
| | | MCA3047 | 3234897 | 3235961 | | | |
| fructose-1,6-bisphosphatase II | fbp2 | Absent | | | Absent | | |
| Transketolase | tkt | MCA3040 | 3226183 | 3228195 | F6R98_00285 | 69185 | 71191 |
| | | MCA3046 | 3232865 | 3234877 | | | |
| fructose-1,6-bisphosphatase I / sedoheptulose-1,7-bisphosphatase | glpX-SEBP | Absent | | | Absent | | |
| Ribose 5-phosphate isomerase A (EC 5.3.1.6) | rpi | MCA0355 | 357582 | 358238 | F6R98_00305 | 74246 | 74902 |

**Supplementary Table S2**. Terminal oxidases encoded in the genomes of strain Shm1, *Mc. capsulatus* Bath and other representative gammaproteobacterial methanotrophs.

| **Microorganism (Genome accession number)** | **Total number of terminal oxidases** | | | |
|---|---|---|---|---|
| | **Low-affinity** | | **Low-/high-affinity** | **High-affinity** |
| | ***$aa_3$*** | ***$bo_3$*** | ***$b(o)a_3/ba_3$*** | ***bd*** |
| Strain Shm1 (CP044205) | F6R98_07605-F6R98_07620 | none | none | F6R98_00185-F6R98_00195, F6R98_03365-F6R98_03375, F6R98_15065-F6R98_15070 |
| *Methylococcus capsulatus* Bath (AE017282.2) | MCA0879-MCA0883 | none | MCA2396-MCA2397 | MCA1105-MCA_RS05455 |
| *Methylomonas methanica* MC09 (NC_015572.1) | Metme_1607-Metme_1609, Metme_3151-Metme_3155 | Metme_1277-Metme_1280 | none | Metme_3309-Metme_3311, Metme_4253-Metme_4254 |
| *Methylomonas* sp. LW13 (NZ_CP033381.1) | U737_RS09955-U737_RS09970 | none | U737_RS18060-U737_RS18065 | U737_RS02705-U737_RS02710, U737_RS11120-U737_RS11130 |
| *Methylobacter tundripaludum* SV96 (AEGW00000000.2) | METTU_RS00670-METTU_RS00685, METTU_RS01055-METTU_RS01060 | none | none | METTU_RS19325-METTU_RS19330, METTU_RS20835-METTU_RS20840 |
| *Methylomicrobium buryatense* 5GB1C (NZ_CP035467.1) | EQU24_RS14110-EQU24_RS14130 | EQU24_08255-EQU24_RS08270 | EQU24_RS09070-EQU24_RS09075 | none |

**Supplementary Table S3**. Genes involved in flagella biosynthesis in the genomes of strain Shm1 and their homologs.

| Start | End | Length | Locus tag | Predicted product | Nearest homolog | E value | AA % Ident | Accession of homolog |
|---|---|---|---|---|---|---|---|---|
| 868702 | 869817 | 1116 | F6R98_03645 | flagellar basal body P-ring protein FlgI | flagellar basal body P-ring protein FlgI [*Sulfuriferula plumbiphila*] | 4,00E-156 | 62.54% | WP_147070396.1 |
| 896848 | 897624 | 777 | F6R98_03780 | flagellar biosynthetic protein FliR | flagellar biosynthetic protein FliR [*Massilia* sp. GV090] | 1,00E-93 | 53.94% | WP_132138946.1 |
| 897715 | 897987 | 273 | F6R98_03785 | flagellar biosynthesis protein FliQ | flagellar biosynthesis protein FliQ [*Massilia glaciei*] | 5,00E-37 | 70.59% | WP_106759030.1 |
| 898087 | 898851 | 765 | F6R98_03790 | The bacterial flagellar biogenesis protein FliP forms a type III secretion system (T3SS)-type pore required for flagellar assembly | flagellar type III secretion system pore protein FliP [*Yersinia mollaretii*] | 5,00E-118 | 74.32% | WP_072079705.1 |
| 898841 | 899203 | 363 | F6R98_03795 | flagellar biosynthetic protein FliO | flagellar biogenesis protein FliO [*Herbaspirillum frisingense* GSF30] | 2,00E-24 | 48.08% | EOA05561.1 |
| 899200 | 899592 | 393 | F6R98_03800 | flagellar motor switch protein FliN | flagellar motor switch protein FliN [*Noviherbaspirillum* sp. UKPF54] | 2,00E-56 | 69.12% | WP_146329939.1 |
| 899582 | 900604 | 1023 | F6R98_03805 | flagellar motor switch protein FliM | flagellar motor switch protein FliM [*Undibacterium* sp. S11R28] | 2,00E-143 | 58.28% | WP_126774426.1 |
| 900619 | 901116 | 498 | F6R98_03810 | flagellar basal body-associated protein FliL | flagellar basal body-associated protein FliL [*Noviherbaspirillum denitrificans*] | 2,00E-37 | 47.33% | OWW18860.1 |
| 901474 | 903438 | 1965 | F6R98_03815 | hypothetical protein | TPA: flagellar hook-length control protein FliK [*Oxalobacteraceae* bacterium] | 3,00E-31 | 46.95% | HCE11017.1 |
| 903451 | 903909 | 459 | F6R98_03820 | flagellar export protein FliJ | flagella biosynthesis chaperone FliJ [*Herbaspirillum robiniae*] | 6,00E-34 | 46.76% | WP_088749909.1 |
| 903906 | 905324 | 1419 | F6R98_03825 | flagellar protein export ATPase FliI | flagellar protein export ATPase FliI [*Herbaspirillum autotrophicum*] | 0 | 71.83% | WP_050462254.1 |
| 905321 | 906031 | 711 | F6R98_03830 | flagellar assembly protein FliH | flagellar assembly protein FliH [*Sulfuriferula thiophila*] | 1,00E-47 | 40.00% | WP_124949960.1 |
| 906024 | 907025 | 1002 | F6R98_03835 | flagellar motor switch protein FliG | flagellar motor switch protein FliG [*Janthinobacterium* sp. 17J80-10] | 3,00E-141 | 60.00% | WP_128901790.1 |
| 907022 | 908776 | 1755 | F6R98_03840 | flagellar basal body M-ring protein FliF | flagellar basal body M-ring protein FliF [*Noviherbaspirillum* sp. 122213-3] | 0 | 55.92% | WP_151636386.1 |
| 908943 | 909308 | 366 | F6R98_03845 | flagellar hook-basal body complex protein FliE | flagellar hook-basal body complex protein FliE [*Methylotenera mobilis*] | 3,00E-39 | 57.02% | WP_015831871.1 |
| 918452 | 918799 | 348 | F6R98_03890 | flagellar biosynthesis protein | flagellar biosynthesis protein [*Solimicrobium silvestre*] | 1,00E-37 | 67.74% | WP_105534246.1 |

| | | | | | | | | |
|---|---|---|---|---|---|---|---|---|
| 918786 | 919829 | 1044 | F6R98_03895 | flagellar hook-length control protein FliK | flagellar hook-length control protein FliK [*Sulfuriferula multivorans*] | 2,00E-49 | 44.98% | WP_124704435.1 |
| 919856 | 920302 | 447 | F6R98_03900 | flagellar protein FliT | flagellar protein FliT [*Burkholderia vietnamiensis*] | 0,068 | 32.29% | WP_059462207.1 |
| 920185 | 920676 | 492 | F6R98_03905 | flagellar export chaperone FliS | flagellar export chaperone FliS [*Undibacterium pigrum*] | 3,00E-48 | 59.09% | WP_110253197.1 |
| 920713 | 922191 | 1479 | F6R98_03910 | flagellar filament capping protein FliD | flagellar filament capping protein FliD [*Tepidiphilus* sp. J10] | 1,00E-70 | 36.86% | WP_142804334.1 |
| 922211 | 922627 | 417 | F6R98_03915 | flagellar protein FlaG | hypothetical protein CVU31_07740 [*Betaproteobacteria* bacterium HGW-*Betaproteobacteria*-4] | 7,00E-11 | 46.97% | PKO47799.1 |
| 922719 | 923924 | 1206 | F6R98_03920 | flagellin | flagellin [*Xanthomonadaceae* bacterium SCN 69-123] | 1,00E-67 | 43.28% | ODU42631.1 |
| 1620403 | 1621341 | 939 | F6R98_07105 | flagellar motor protein MotB | flagellar motor protein MotB [*Sulfuriferula* sp. AH1] | 4,00E-124 | 59.79% | WP_087447141.1 |
| 1621358 | 1622212 | 855 | F6R98_07110 | flagellar motor stator protein MotA | flagellar motor stator protein MotA [*Herbaspirillum autotrophicum*] | 2,00E-162 | 77.82% | WP_050461989.1 |
| 1623205 | 1623612 | 408 | F6R98_07120 | flagellar basal body rod protein FlgC | flagellar basal body rod protein FlgC [*Herbaspirillum autotrophicum*] | 3,00E-55 | 63.16% | WP_050462273.1 |
| 1623618 | 1623983 | 366 | F6R98_07125 | flagellar basal body rod protein FlgB | flagellar basal body rod protein FlgB [*Sulfuriferula multivorans*] | 2,00E-41 | 54.07% | WP_124703299.1 |
| 1624140 | 1624859 | 720 | F6R98_07130 | flagellar basal body P-ring formation protein FlgA | flagellar basal body P-ring formation protein FlgA [*Herbaspirillum* sp. CF444] | 3,00E-59 | 41.95% | WP_007882620.1 |
| 1624964 | 1625293 | 330 | F6R98_07135 | flagellar biosynthesis anti-sigma factor FlgM | flagellar biosynthesis anti-sigma factor FlgM [*Duganella* sp. Leaf126] | 7,00E-12 | 42.59% | WP_056157072.1 |
| 1625295 | 1625789 | 495 | F6R98_07140 | flagellar protein FlgN | flagellar protein FlgN [*Paucimonas lemoignei*] | 2,00E-20 | 36.60% | WP_132256316.1 |
| 1625779 | 1626834 | 1056 | F6R98_07145 | OmpA family protein | flagellar motor protein MotD [*Sulfuriferula multivorans*] | 1,00E-70 | 41.77% | WP_124706240.1 |
| 1626845 | 1627591 | 747 | F6R98_07150 | flagellar motor protein | flagellar motor protein [*Noviherbaspirillum denitrificans*] | 2,00E-95 | 56.15% | WP_088708245.1 |
| 1627594 | 1628307 | 714 | F6R98_07155 | RNA polymerase sigma factor FliA | flagellar motor protein [*Noviherbaspirillum denitrificans*] | 2,00E-95 | 56.15% | WP_088708245.1 |
| 1628311 | 1629093 | 783 | F6R98_07160 | MinD/ParA family protein | antiactivator of flagellar biosynthesis FleN protein [*Massilia* sp. K1S02-61] | 1,00E-63 | 44.84% | WP_119810355.1 |
| 1629090 | 1630367 | 1278 | F6R98_07165 | flagellar biosynthesis protein FlhF | flagellar biosynthesis protein FlhF [*Sulfuriferula plumbiphila*] | 5,00E-142 | 51.92% | WP_147070425.1 |
| 1630364 | 1632448 | 2085 | F6R98_07170 | flagellar biosynthesis protein FlhA | TPA: flagellar biosynthesis protein FlhA [*Betaproteobacteria* bacterium] | 0 | 75.47% | HAN56499.1 |

| | | | | | | | | |
|---|---|---|---|---|---|---|---|---|
| 1632644 | 1633783 | 1140 | F6R98_07175 | flagellar type III secretion system protein FlhB | flagellar type III secretion system protein FlhB [*Massilia* sp. Leaf139] | 1,00E-151 | 57.57% | WP_056341970.1 |
| 1634869 | 1636434 | 1566 | F6R98_07185 | flagellar hook-basal body complex protein | flagellar hook protein FlgE [*Gallionellaceae* bacterium] | 1,00E-32 | 31.96% | TNC98404.1 |
| 1636704 | 1637441 | 738 | F6R98_07190 | flagellar basal body rod protein FlgF | MULTISPECIES: flagellar basal-body rod protein FlgF [unclassified *Herbaspirillum*] | 2,00E-90 | 56.91% | WP_141915740.1 |
| 1637554 | 1638342 | 789 | F6R98_07195 | flagellar basal-body rod protein FlgG | flagellar basal-body rod protein FlgG [Herbaspirillum autotrophicum] | 5,00E-117 | 64.89% | WP_050462269.1 |
| 1638432 | 1639106 | 675 | F6R98_07200 | flagellar basal body L-ring protein FlgH | flagellar basal body L-ring protein FlgH [*Thiobacillus* sp.] | 1,00E-57 | 45.66% | TXH73323.1 |
| 1639115 | 1640236 | 1122 | F6R98_07205 | flagellar basal body P-ring protein FlgI | flagellar basal body P-ring protein FlgI [*Sulfuriferula plumbiphila*] | 2,00E-165 | 64.21% | WP_147070396.1 |
| 1640239 | 1641111 | 873 | F6R98_07210 | flagellar assembly peptidoglycan hydrolase FlgJ | flagellar assembly peptidoglycan hydrolase FlgJ [*Sulfuriferula thiophila*] | 5,00E-102 | 54.45% | WP_124949226.1 |
| 1641185 | 1643824 | 2640 | F6R98_07215 | flagellar hook-associated protein FlgK | flagellar hook-associated protein FlgK [*Sulfuriferula plumbiphila*] | 2,00E-86 | 38.36% | WP_147070392.1 |
| 1643839 | 1644825 | 987 | F6R98_07220 | flagellar hook-associated protein 3 | flagellar hook-associated protein 3 [*Sulfuriferula multivorans*] | 4,00E-55 | 39.33% | WP_124703289.1 |
| 3470390 | 3470785 | 396 | F6R98_15745 | hypothetical protein | flagellin domain-containing protein [*Hylemonella gracilis* ATCC 19624] | 4,00E-22 | 46.72% | EGI78503.1 |

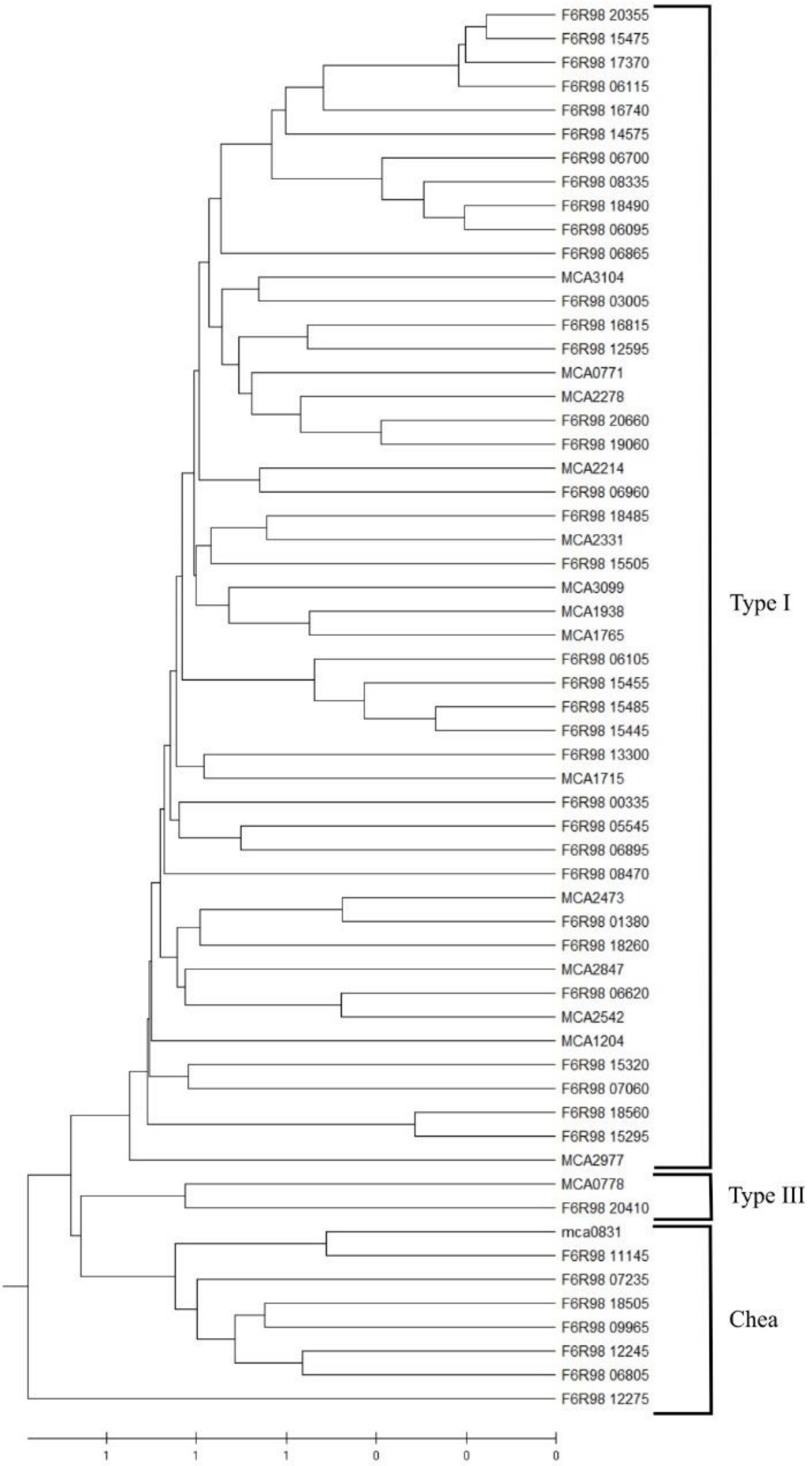


**Figure S1.** Relationship between the deduced amino acid sequences of HK genes retrieved from the genomes of *M. capsulatus Bath* and strain Shm1. The tree was constructed using MEGAX with UPGMA as hierarchical cluster method. The scale indicates genetic distance between amino acid sequences.

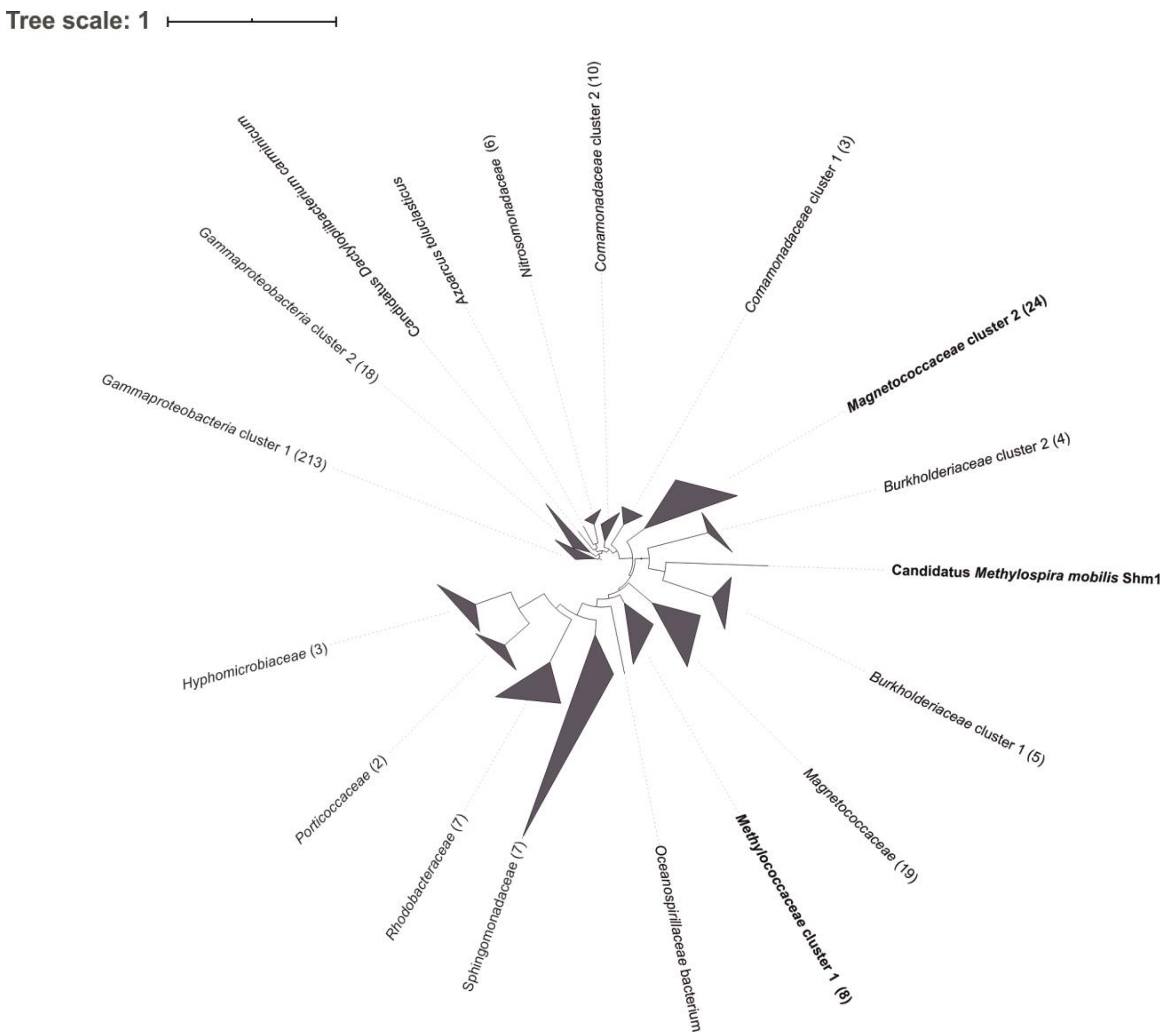


**Figure S2.** Phylogenetic position of the FlaB protein from strain Shm1. The tree was constructed based on 235 amino acid residues of flagellin-encoded proteins using the Fast Tree package and approximately maximum-likelihood method. Bar, 1 substitutions per amino acid position.
